# Supplementary material for: Allosteric modulation of the CXCR4:CXCL12 axis by targeting receptor nanoclustering via the TMV-TMVI domain
Source: eLife. 2024 Sep 9;13:RP93968. doi: 10.7554/eLife.93968 (PMC11383527; doi:10.7554/eLife.93968)
Supplement: Figure 1—source data 1. — Structure of the compounds employed in the study. The compounds studied in detail (AGR1.131, AGR1.135 and AGR1.137) are highlighted in green boxes. The percentage of inhibition of cell migration in response to CXCL12 is also shown. [file elife-93968-fig1-data1.docx]

**Figure 1 – Source Data 1**. Compounds with minimal interaction energy in the area of interest

| Ref. | Structure | Ref. | Structure |
| --- | --- | --- | --- |
| #1  AGF2.9C1  % Inh: 38.01 |  | #15  LNR1.17  % Inh: 48.71 |  |
| #2  AGF2.6  % Inh: 12.88 |  | #16  VP1.23  % Inh: 30.69 |  |
| #3  VP4.29  % Inh: 0 |  | #17  AGR1.138  % Inh: 45.68 |  |
| #4  MR4.17  % Inh: 3.43 |  | #18  AGR1.103  % Inh: 53.55 |  |
| #5  AGR1.209  % Inh: 1.49 |  | #19  AGR1.135  % Inh: 65.90 |  |
| #6  AGR1.101  % Inh: 36.19 |  | #20  JHD1.47  % Inh: 11.79 |  |
| #7  MR3.24  % Inh: 2.14 |  | #21  JAR3.8  % Inh: 21.03 |  |
| #8  VP4.27  % Inh: 0 |  | #22  VNG1.61  % Inh: 0 |  |
| #9  MR2.57  % Inh: 0 |  | #23  AGR1.210  % Inh: 40.15 |  |
| #10  AGR1.131  % Inh: 2.34 |  | #24  AGF351  % Inh: 21.23 |  |
| #11  MR1.50  % Inh: 40.91 |  | #25  IGS-2.73  % Inh: 27.36 |  |
| #12  AGR1.117  % Inh: 31.74 |  | #26  VNG1.64  % Inh: 14.31 |  |
| #13  VSP3.1  % Inh: 36.43 |  | #27  DM1.27  % Inh: 28.54 |  |
| #14  VP1.11  % Inh: 28.07 |  | #28  ERP3.1  % Inh: 37.06 |  |
| Ref. | Structure | Ref. | Structure |
| #29  ERP2.51  % Inh: 52.50 |  | #35  SC089  % Inh: 0.98 |  |
| #30  AGR1.267  % Inh: 30.06 |  | #36  AGR1.137  % Inh: 89.82 |  |
| #31  VNG1.63  % Inh: 19.98 |  | #37  IGS-4.32  % Inh: 0 |  |
| #32  AGR1.134  % Inh: 23.65 |  | #38  IGS-2.3  % Inh: 0 |  |
| #33  SC513  % Inh: 12.94 |  | #39  MR2.2  % Inh: 0 |  |
| #34  SC523  % Inh: 1.37 |  | #40  JAR229  % Inh: |  |

Structure of the compounds employed in the study. The compounds studied in detail (AGR1.131, AGR1.135 and AGR1.137) are highlighted in green boxes. The percentage of inhibition of cell migration in response to CXCL12 is also shown.
