# Supplementary material for: Allosteric modulation of the CXCR4:CXCL12 axis by targeting receptor nanoclustering via the TMV-TMVI domain
Source: eLife. 2024 Sep 9;13:RP93968. doi: 10.7554/eLife.93968 (PMC11383527; doi:10.7554/eLife.93968)
Supplement: Figure 1—source data 2. [file elife-93968-fig1-data2.docx]

**Figure 1 – Source Data 2. Compound synthesis and characterization**

**1. General Experimental Details**

**2. Synthesis and Characterization** **of Triazol derivatives**

**3.** **NMR Spectra and HRMS Analysis**

**1. General Experimental Details**

All solvents were purchased from Sigma-Aldrich (anhydrous solvents) and commercially available reagents were used as received. All reactions were followed by TLC analysis (TLC plates GF254, Merck) or LC–MS (liquid chromatography mass spectrometry). Melting points were determined in a Büchi Melting Point M-560 apparatus. NMR spectra were recorded at ambient temperature unless otherwise stated using standard pulse methods on one of the following spectrometers and signal frequencies: Bruker AV-300 (1H = 300 MHz, 13C = 75 MHz). Chemical shifts are reported in ppm and are referenced to tetramethylsilane (TMS) or to the following solvent peaks: CDCl3 (1H = 7.27 ppm, 13C = 77.00 ppm), DMSO-d6 (1H = 2.50 ppm, 13C = 39.51 ppm). Coupling constants are quoted to the nearest 0.1 Hz, and multiplicities are given by the following abbreviations and combinations thereof: s (singlet), d (doublet), t (triplet), q (quartet), m (multiplet), and br (broad). Column chromatography was performed on prepacked silica gel columns using a Biotage SP4 or Isolera One system. High resolution mass spectra (HRMS-ESI) were recorded on a Micromass Q-Tof Ultima hybrid quadrupole time-of-flight mass spectrometer, with analytes separated on an Agilent 1100 liquid chromatograph equipped with a Phenomenex Luna C18(2) reversed phase column (100 mm × 2.1 mm, 3 μm packing diameter).

**2. Synthesis and Characterization** **of Triazol derivatives**

**General Procedure (GP):**

Over a solution of 4-((4-(4-(hydroxymethyl)phenyl)-1H-1,2,3-triazol-1-yl)methyl) benzoic acid (123 mg, 0.4 mmol) in 4 ml of DMF, DMAP (97 mg, 0.8 mmol) and EDC.HCl (92 mg, 0.48 mmol) were added and stirred for 15 min at room temperature. Subsequently, the corresponding amine (0.4 mmol) was added and stirred at room temperature for 48 hrs. Next, H_2_O (10 ml) was added and extracted with DCM (25 ml × 3). The organic phase was dried over MgSO_4_, filtered and evaporated under reduced pressure. The crude sample obtained was purified by column chromatography, eluting with a DCM-MeOH solvent mixture (from 99:1 to 90:10).

***N*-Benzyl-4-((4-(4-(hydroxymethyl)phenyl)-1H-1,2,3-triazol-1-yl)methyl)benzamide (AGR1.131)**

The reaction was performed according to GP using benzylamine (44 μl, 0.4 mmol), and the title product was obtained after purification by silica gel column chromatography as a white solid (35% yield). Pf: 193 – 195 ºC.

^1^H NMR (300 MHz, DMSO-*d*_6_) δ 9.09 (t, *J* = 6.0 Hz, 1H), 8.65 (s, 1H), 7.98 – 7.89 (m, 2H), 7.86 – 7.78 (m, 2H), 7.46 (d, *J* = 8.2 Hz, 2H), 7.40 (d, *J* = 8.1 Hz, 2H), 7.36 – 7.20 (m, 5H), 5.73 (s, 2H), 5.25 (t, *J* = 5.7 Hz, 1H), 4.53 (d, *J* = 5.7 Hz, 2H), 4.49 (d, *J* = 6.0 Hz, 2H). ^13^C NMR (75 MHz, DMSO) δ 165.69, 146.69, 142.29, 139.56, 139.01, 134.08, 128.99, 128.25, 127.83, 127.74, 127.12, 126.88, 126.71, 124.90, 121.42, 62.61, 52.58, 42.56.

HRMS (ESI^+^) m/z Calc. for C_24_H_23_N_4_O_2_ [M+H] ^+^: 399.1816. Found: 399.1809.

**(4-((4-(4-(Hydroxymethyl)phenyl)-1H-1,2,3-triazol-1-yl)methyl)phenyl)(4-(2-nitro-4-(trifluoromethyl)phenyl)piperazin-1-yl)methanone (AGR1.135)**

The reaction was performed according to GP using (2-nitro-4-(trifluoromethyl)phenyl)piperazine (110 mg, 0.4 mmol), and the title product was obtained after purification by silica gel column chromatography as a yellow solid (37% yield). Pf: 184 – 186 ºC.

^1^H NMR (300 MHz, DMSO-*d*_6_) δ 8.65 (s, 1H), 8.19 (d, *J* = 2.3 Hz, 1H), 7.89 (dd, *J* = 8.9, 2.3 Hz, 1H), 7.81 (d, *J* = 8.0 Hz, 2H), 7.53 – 7.35 (m, 7H), 5.71 (s, 2H), 5.23 (t, *J* = 5.7 Hz, 1H), 4.52 (d, *J* = 5.4 Hz, 2H), 3.73 (s, 2H), 3.48 (s, 2H), 3.21 (s, 4H). ^13^C NMR (75 MHz, DMSO) δ 169.12, 147.70, 147.08, 142.67, 140.04, 137.84, 135.74, 130.59, 130.54, 129.38, 128.27, 128.00, 127.25, 125.65, 125.29, 124.12, 124.07, 122.06, 121.92, 121.82, 120.57, 120.12, 62.99, 62.87, 52.97, 50.33, 47.01.

HRMS (ESI^+^) Calc. for C_28_H_26_F_3_N_6_O_4_ [M+H] ^+^: 567.1962. Found: 567.1959.

**Ethyl 1-(4-((4-(4-(hydroxymethyl)phenyl)-1H-1,2,3-triazol-1-yl)methyl)benzoyl) piperidine-4-carboxylate (AGR1.137)**

The reaction was performed according to GP using ethyl piperidinee-4-carboxylate (63 mg, 0.4 mmol) and the title product was obtained after purification by silica gel column chromatography as a white solid (30% yield). Pf: 119 – 121 ºC.

^1^H NMR (300 MHz, Methanol-*d*_4_) δ 8.40 (s, 1H), 7.89 – 7.74 (m, 2H), 7.56 – 7.31 (m, 6H), 5.73 (s, 2H), 4.66 (s, 2H), 4.48 (d, *J* = 13.2 Hz, 1H), 4.16 (q, *J* = 7.1 Hz, 2H), 3.69 (d, *J* = 13.0 Hz, 1H), 3.24 – 2.96 (m, 2H), 2.78 – 2.52 (m, 1H), 2.15 – 1.52 (m, 4H), 1.27 (t, *J* = 7.1 Hz, 3H). ^13^C NMR (75 MHz, MeOD) δ 175.82, 171.87, 149.21, 143.15, 138.81, 137.25, 130.56, 129.43, 128.59, 128.56, 126.71, 122.39, 64.88, 61.79, 54.58, 42.65, 41.96, 29.67, 28.96, 14.51.

HRMS (ESI^+^) Calc. For C_25_H_29_N_4_O_4_ [M+H] ^+^: 449.2183. Found: 449.2160.

**3.** **NMR Spectra and HRMS Analysis**

**(AGR1.131)**

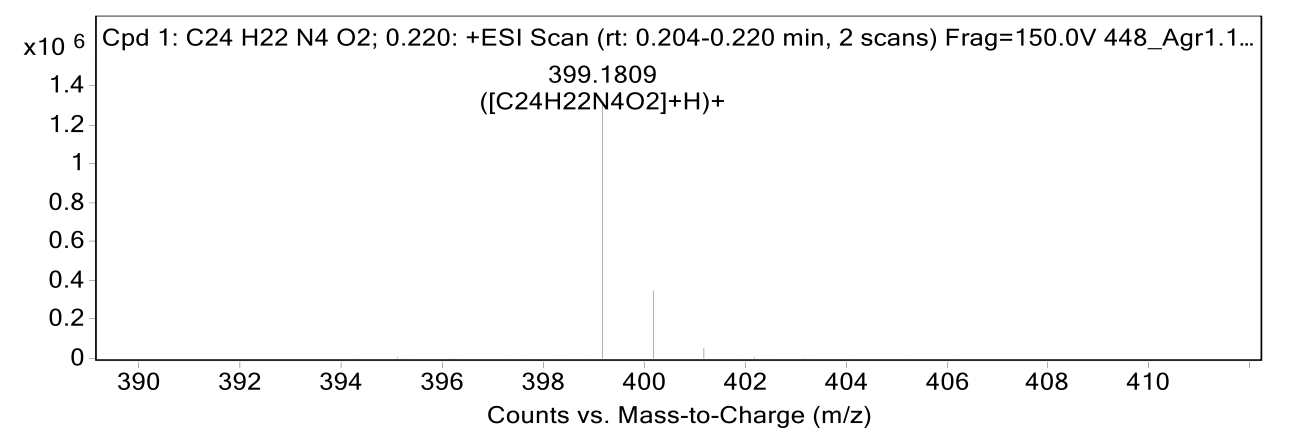


**(AGR1.135)**

**
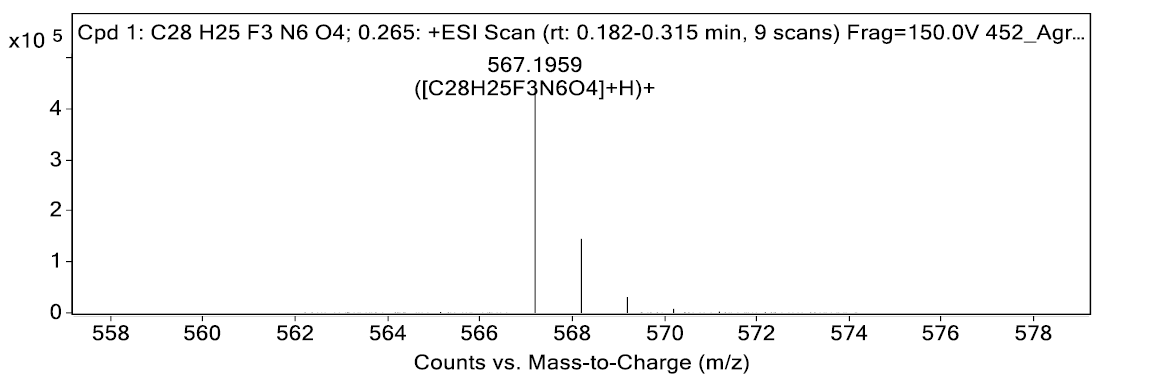
**

**(AGR1.137)**

**
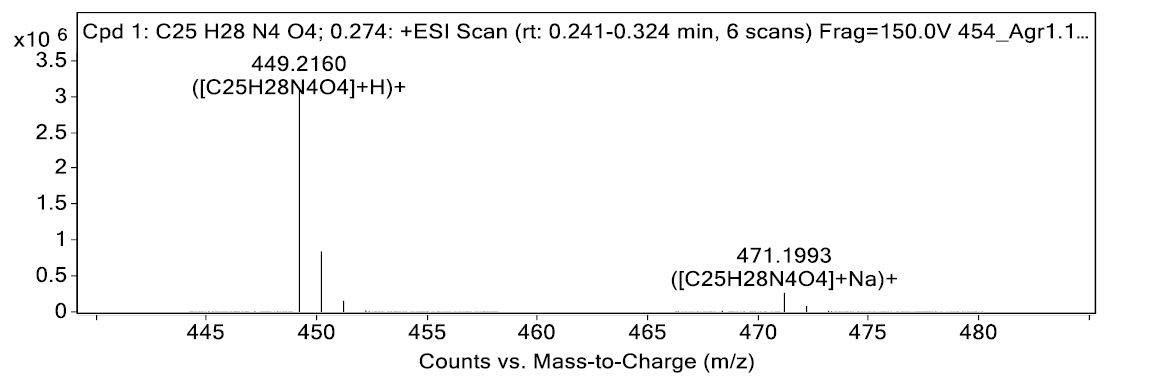
**
