# Supplementary material for: Allosteric modulation of the CXCR4:CXCL12 axis by targeting receptor nanoclustering via the TMV-TMVI domain
Source: eLife. 2024 Sep 9;13:RP93968. doi: 10.7554/eLife.93968 (PMC11383527; doi:10.7554/eLife.93968)
Supplement: Figure 3—source data 1. [file elife-93968-fig3-data1.zip › Figure 3_Source Data 1.pdf]

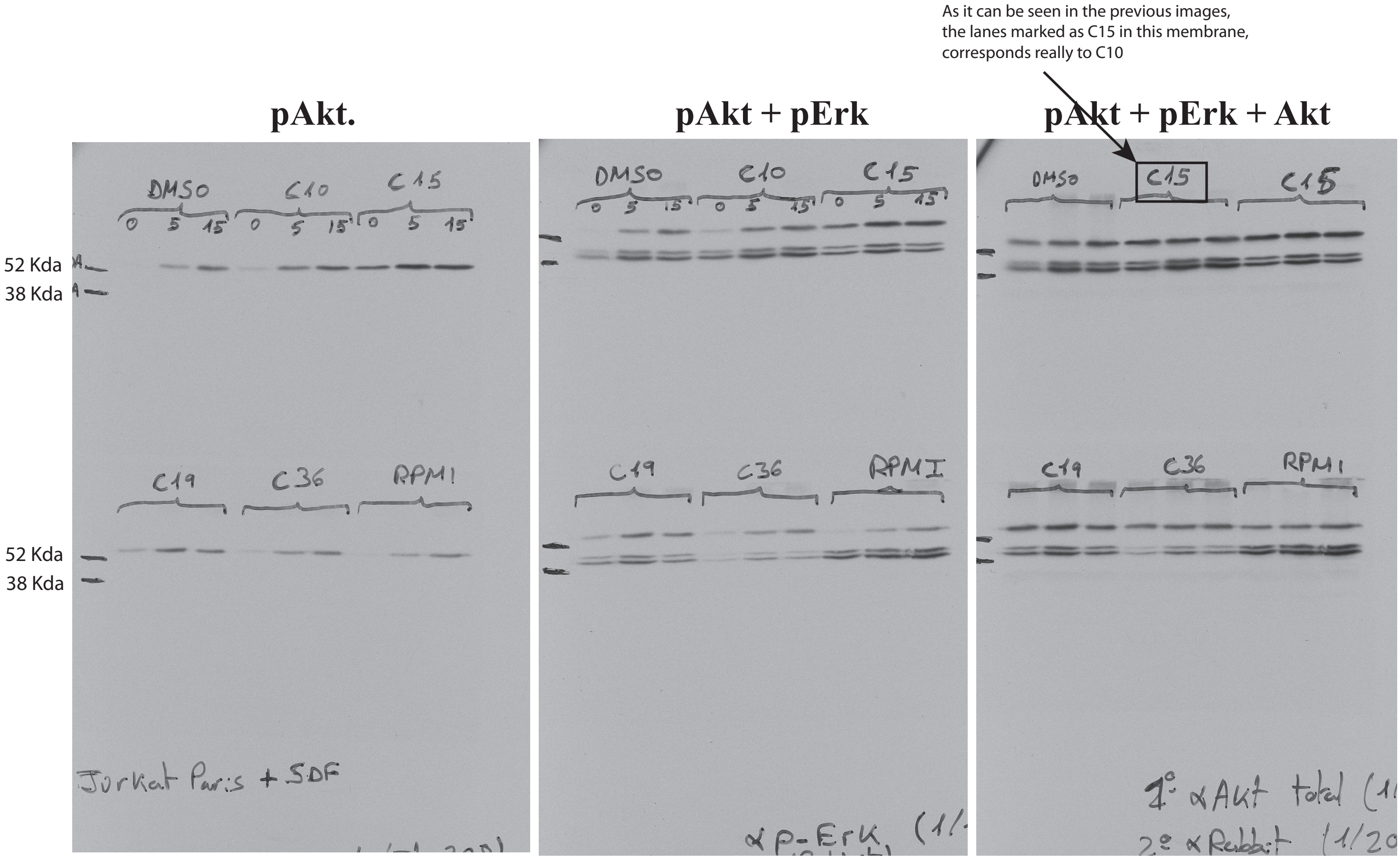

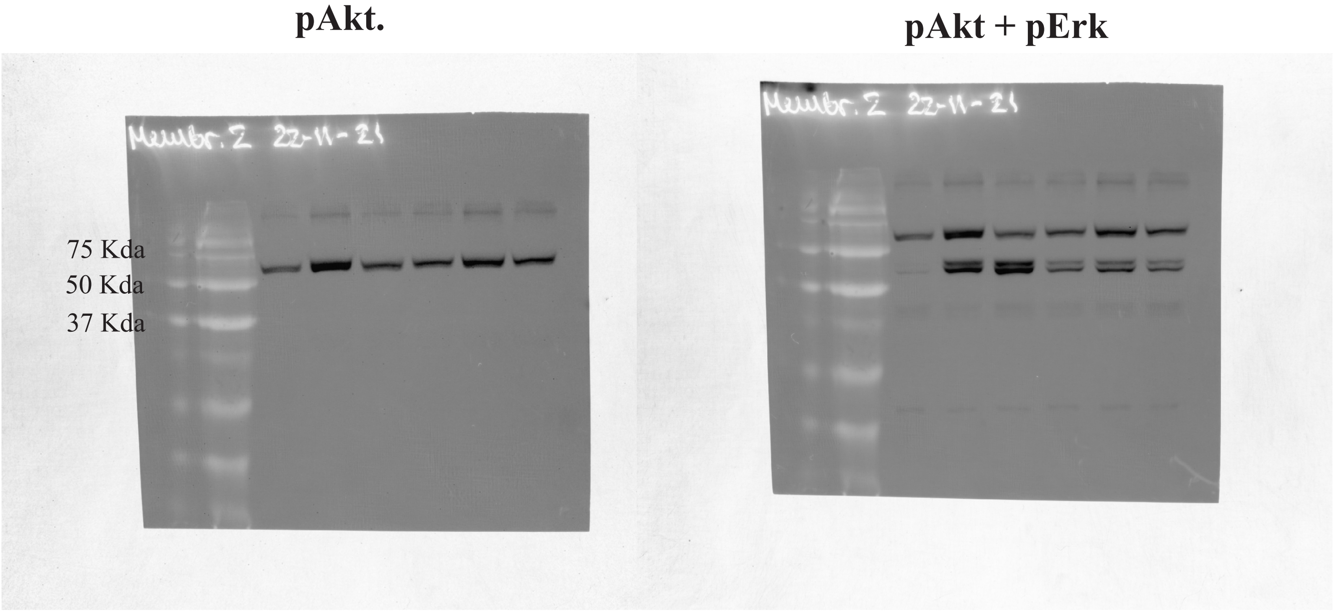

Figure 3, Source Data 1. Original membranes corresponding to Figure 3, panel D. C10 and C19 are our internal codes for AGR1.131 and AGR1.135, respectively. Rainbow molecular weight markers were employed. The lower membranes correspond to AGR1.137-treated cells (first three lanes) and DMSO control (lanes 4, 5 and 6; not shown in figure 3) and usin all blue molecular weight markers.
