# Supplementary figures and images for: Allosteric modulation of the CXCR4:CXCL12 axis by targeting receptor nanoclustering via the TMV-TMVI domain
Source: eLife. 2024 Sep 9;13:RP93968. doi: 10.7554/eLife.93968 (PMC11383527; doi:10.7554/eLife.93968)

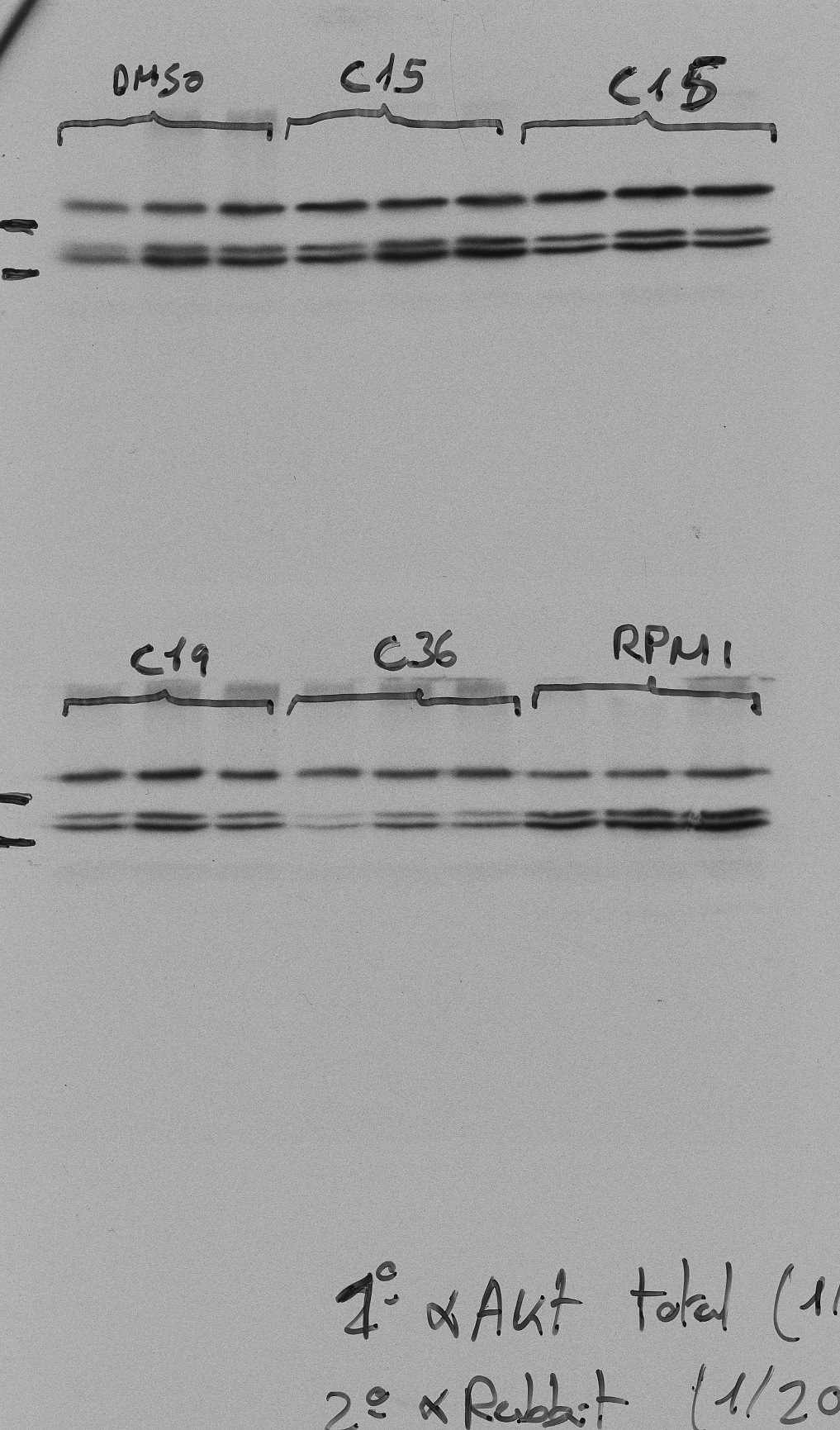

Supplement: Figure 3—source data 2. [file elife-93968-fig3-data2.zip › Figure 3_Source data 2/Akt total.tif]

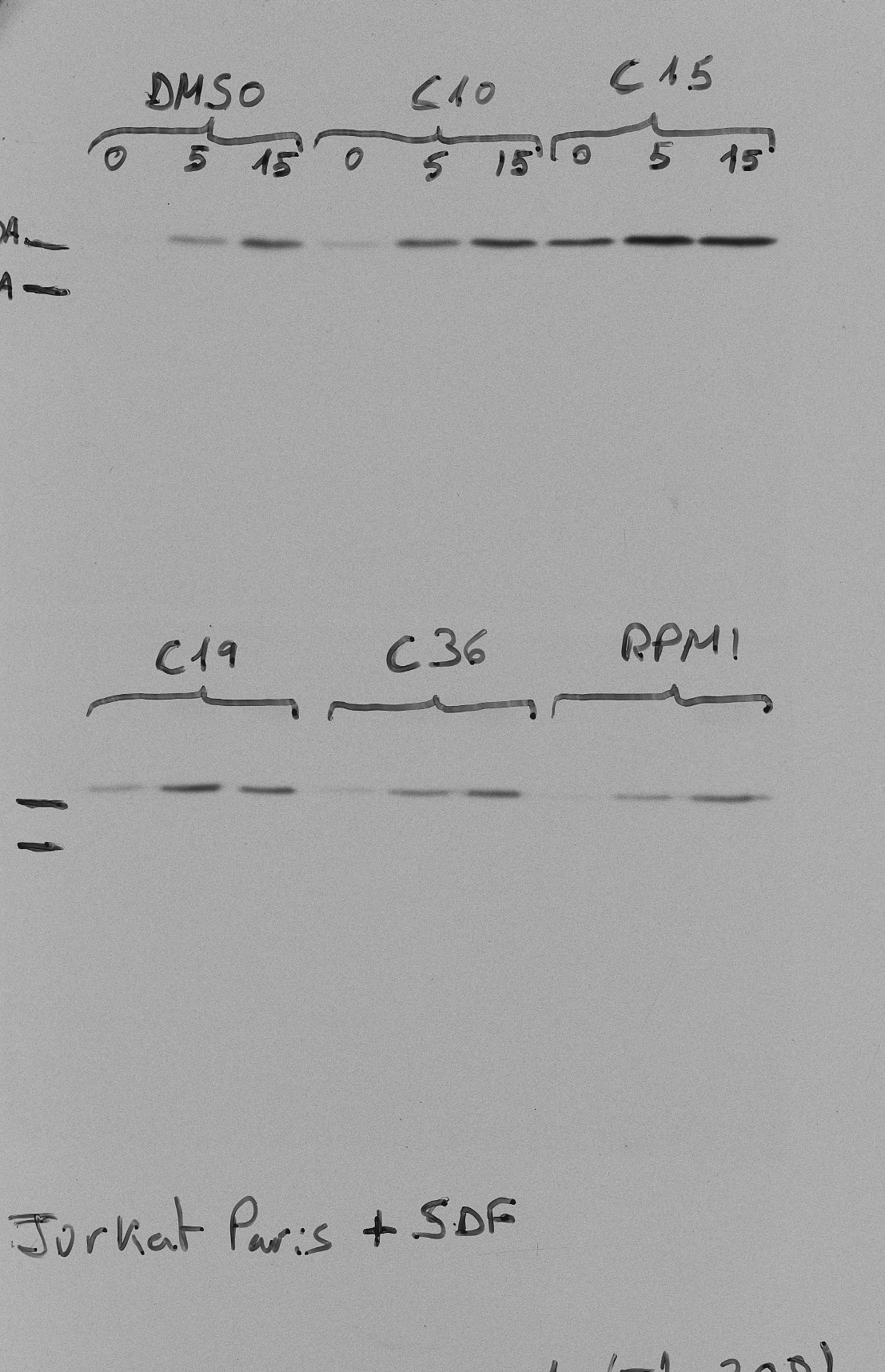

Supplement: Figure 3—source data 2. [file elife-93968-fig3-data2.zip › Figure 3_Source data 2/P-Akt215.tif]

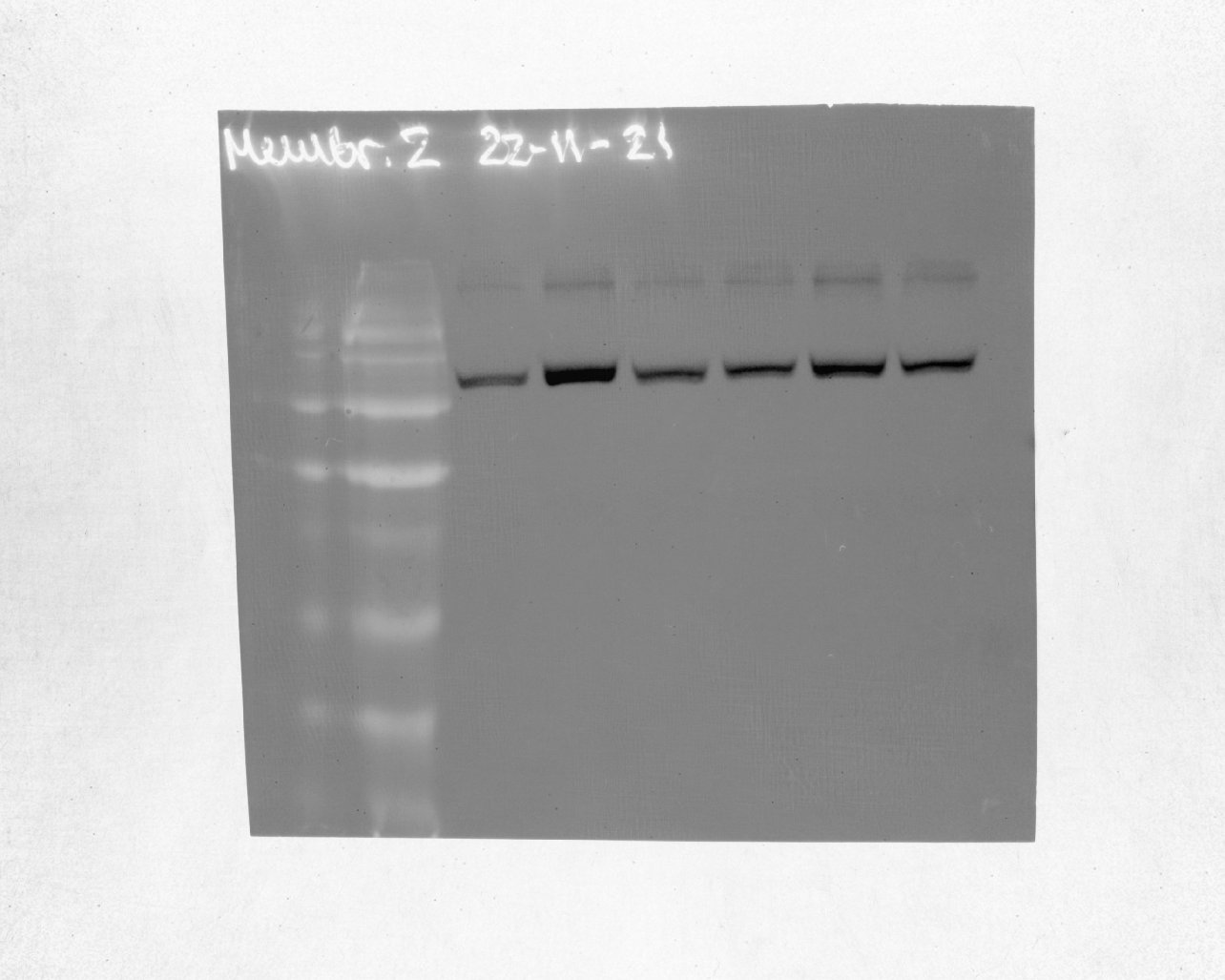

Supplement: Figure 3—source data 2. [file elife-93968-fig3-data2.zip › Figure 3_Source data 2/416 MM 2021-11-23 13h16m32s(Composite).tif]

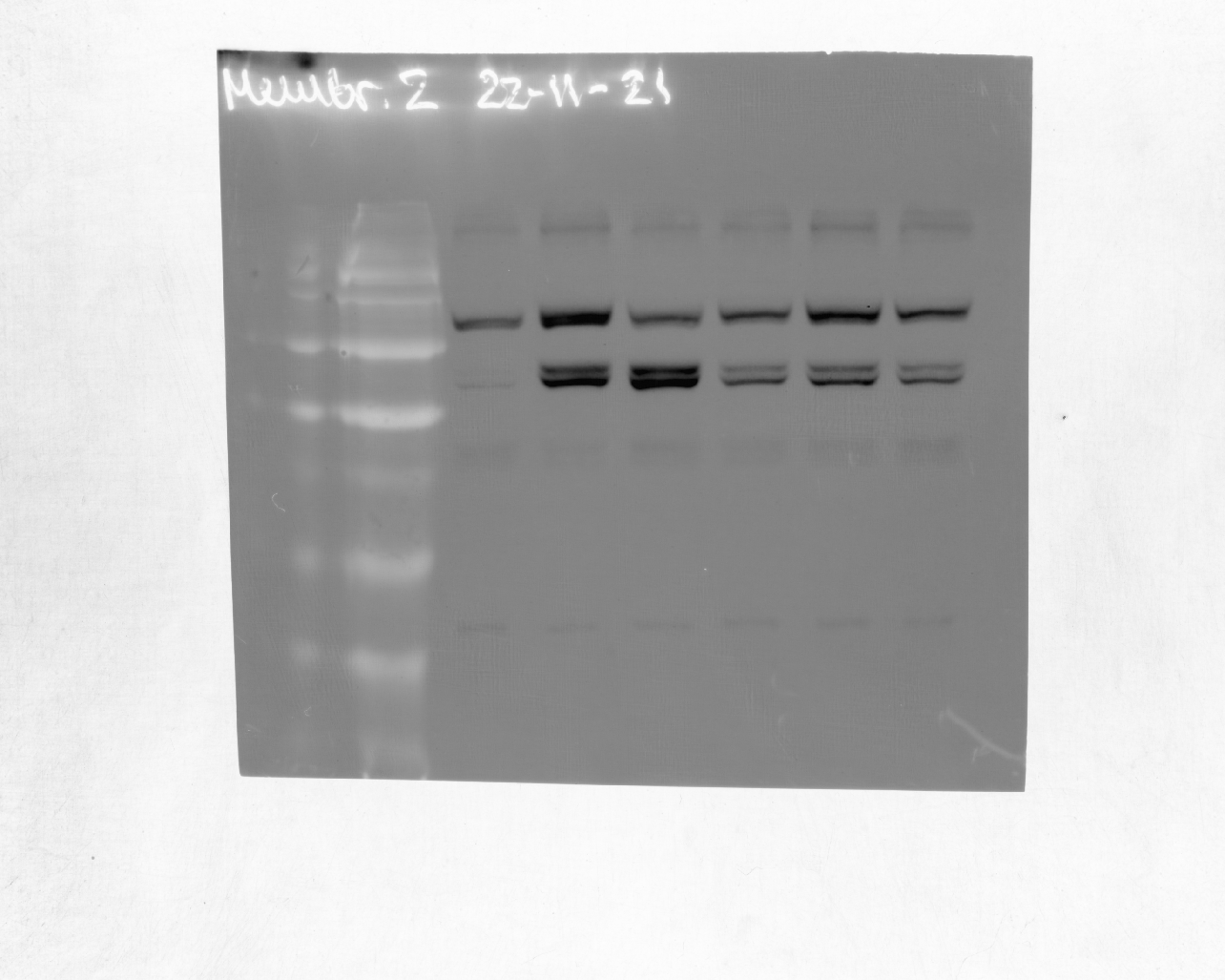

Supplement: Figure 3—source data 2. [file elife-93968-fig3-data2.zip › Figure 3_Source data 2/416 MM 2021-11-24 13h48m31s(Composite).tif]

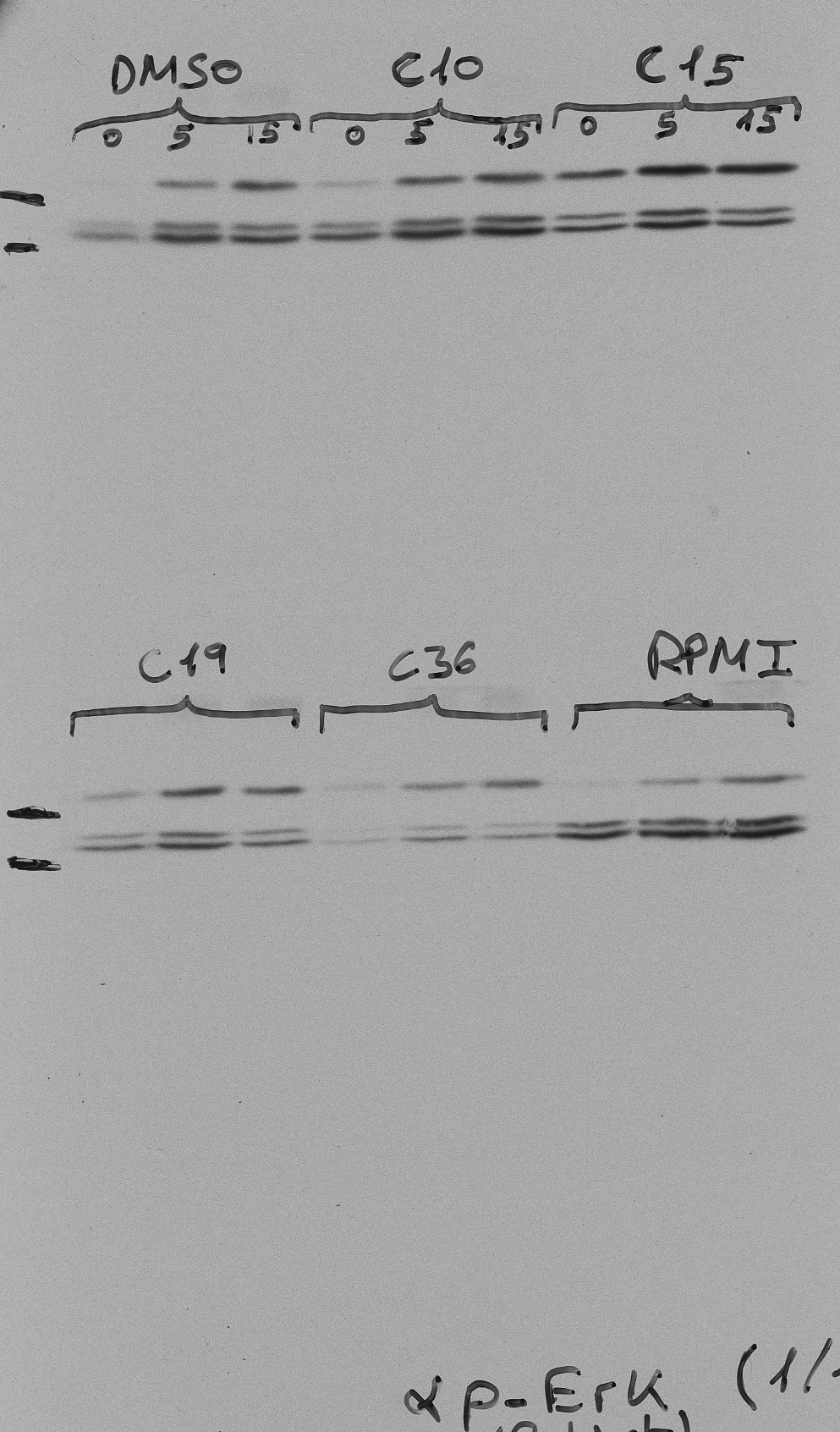

Supplement: Figure 3—source data 2. [file elife-93968-fig3-data2.zip › Figure 3_Source data 2/P-Erk213.tif]

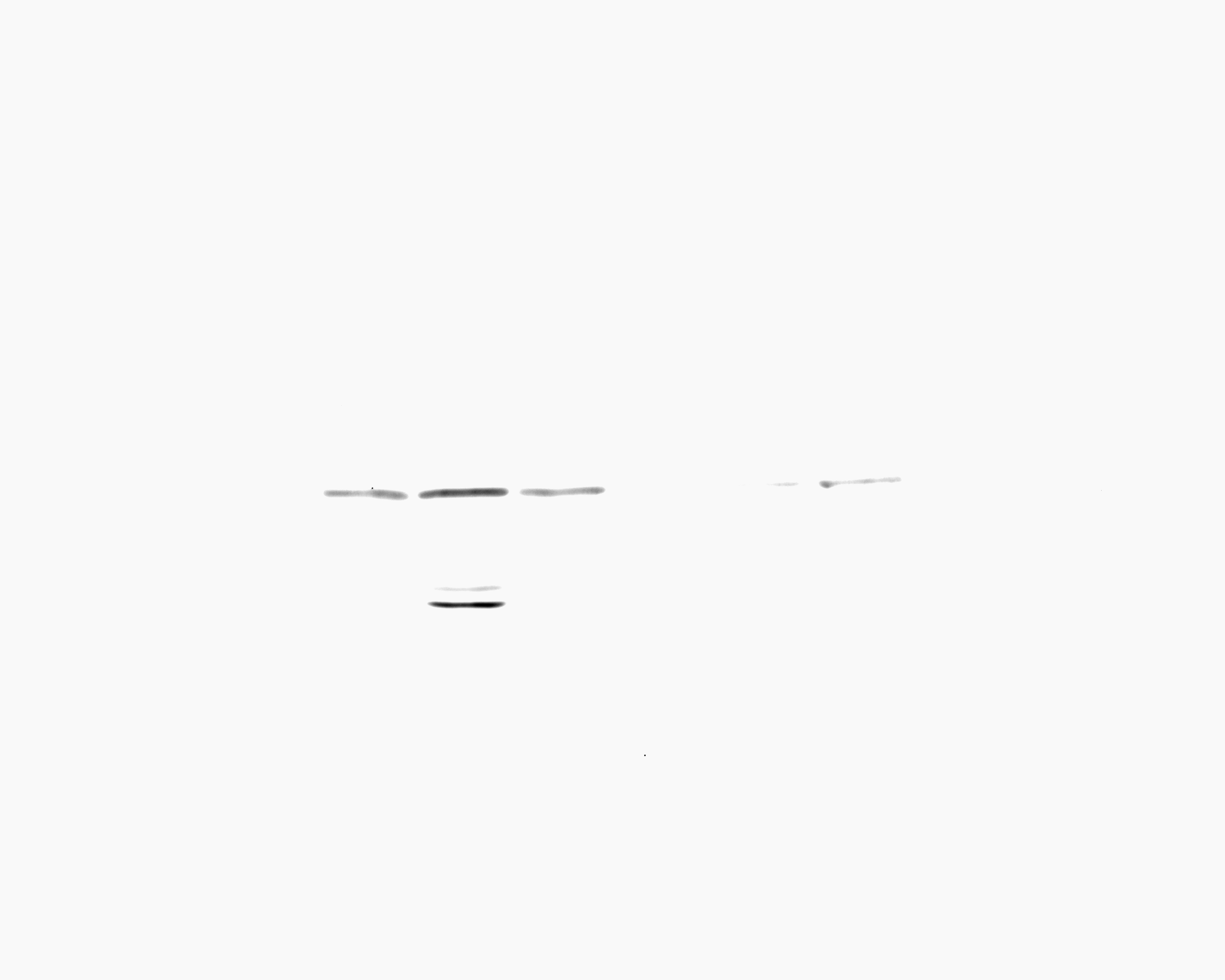

Supplement: Figure 3—figure supplement 2—source data 2. [file elife-93968-fig3-figsupp2-data2.zip › Figure 3_ Figure supplement 2_ Source data 2/416 MM 2024-03-14 16h48m20s(StarBright B700)alternative.raw16.tif]

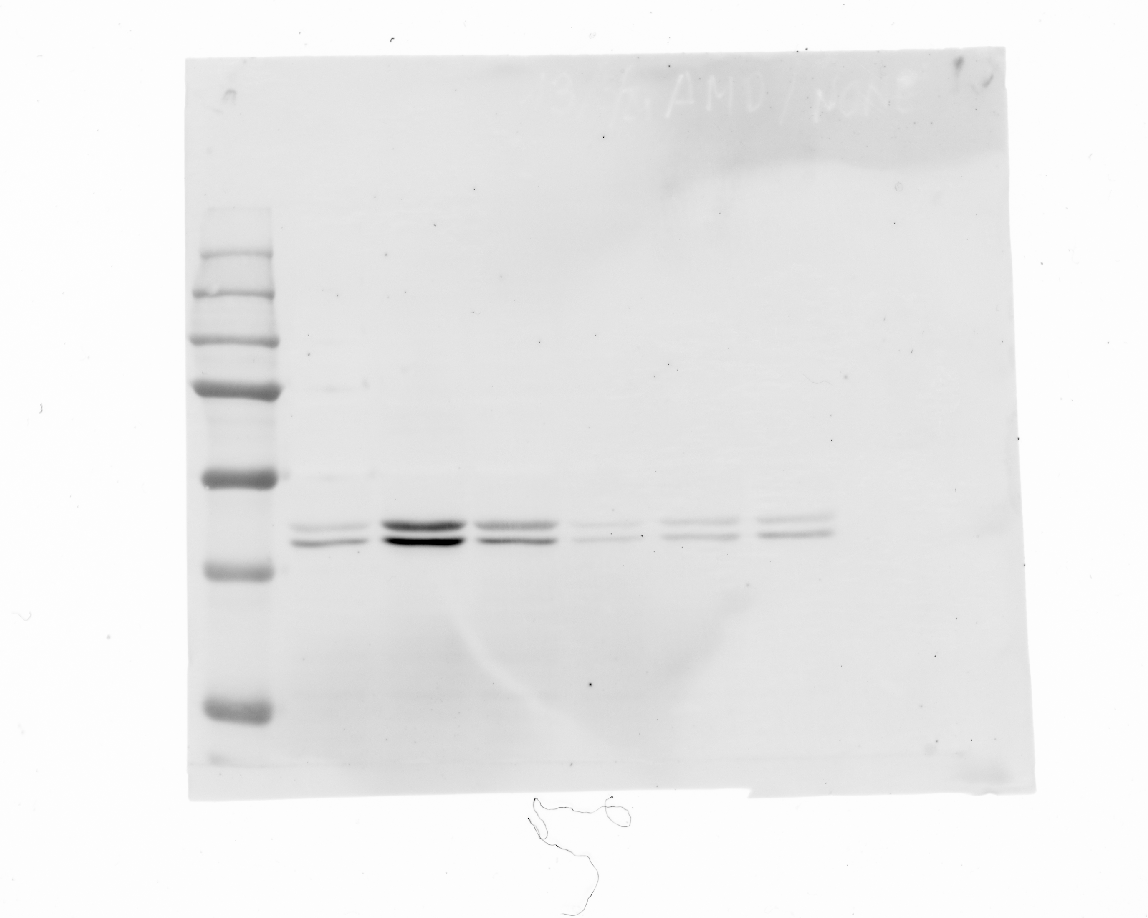

Supplement: Figure 3—figure supplement 2—source data 2. [file elife-93968-fig3-figsupp2-data2.zip › Figure 3_ Figure supplement 2_ Source data 2/416 MM 2024-03-14 11h16m46s(StarBright B700).tif]

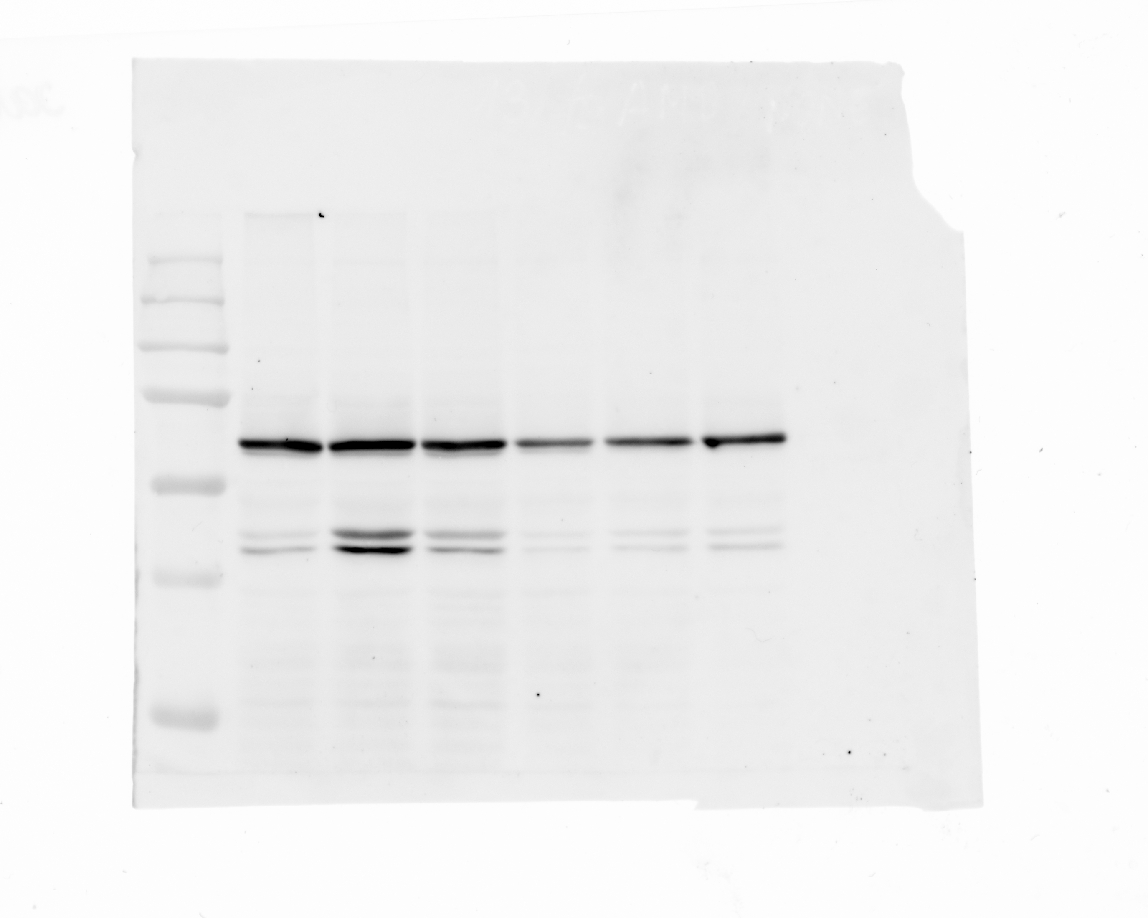

Supplement: Figure 3—figure supplement 2—source data 2. [file elife-93968-fig3-figsupp2-data2.zip › Figure 3_ Figure supplement 2_ Source data 2/416 MM 2024-03-15 13h37m55s(StarBright B700).jpg]
