# Supplementary material for: Allosteric modulation of the CXCR4:CXCL12 axis by targeting receptor nanoclustering via the TMV-TMVI domain
Source: eLife. 2024 Sep 9;13:RP93968. doi: 10.7554/eLife.93968 (PMC11383527; doi:10.7554/eLife.93968)
Supplement: Figure 3—figure supplement 2—source data 1. [file elife-93968-fig3-figsupp2-data1.zip › Figure 3_Figure supplement 2_Source data 1.pdf]

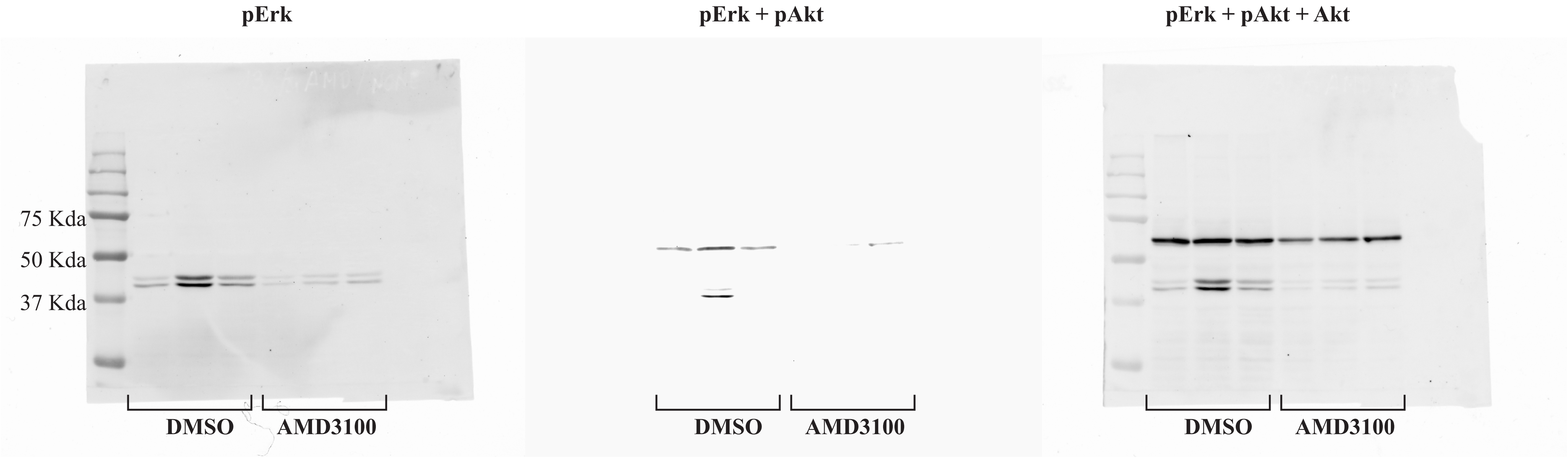

Figure 3, Figure supplement 2, Source Data 1. Original membranes corresponding to Figure supplement 2. First three lanes correspond to DMSO-treated cells and lanes 4, 5 and 6 to cells treated with AMD-3100. All blue molecular weight markers were employed.
